# Supplementary material for: Fasting alters the gut microbiome reducing blood pressure and body weight in metabolic syndrome patients
Source: Nat Commun. 2021 Mar 30;12:1970. doi: 10.1038/s41467-021-22097-0 (PMC8010079; doi:10.1038/s41467-021-22097-0)
Supplement: Supplementary file 1 — Supplementary Information [file 41467_2021_22097_MOESM1_ESM.pdf]

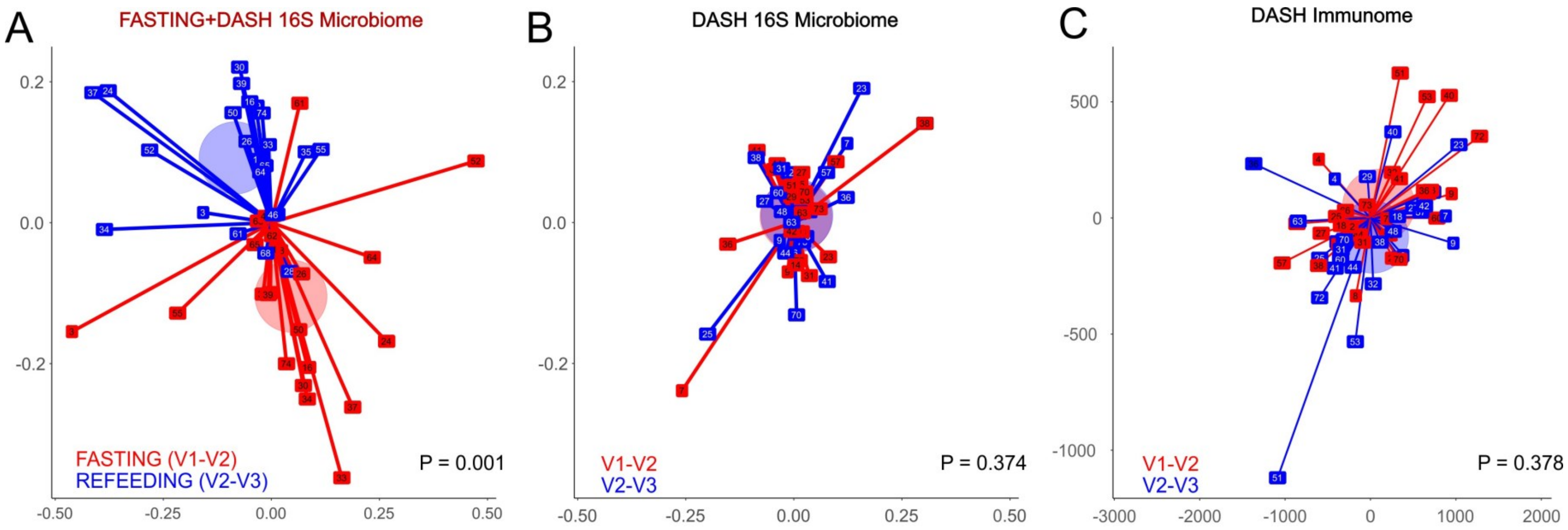

Supplementary Fig. 1. Changes to the microbiome at the level of 16S OTUs in the fasting+DASH and DASH groups, and changes to the immunome in the DASH arm. Unconstrained Principal Coordinates graph with first two dimensions shown. Axes show fasting and refeeding deltas in the case of fasting+DASH (A) and DASH, and V1-V2, V2-V3 deltas in the case of DASH after one-week intervention and three-month. Pseudonym participant ID numbers are shown on the point markers. Transparent circle markers show arithmetic mean position of one week intervention and three-month (A, B). Circles of (C) denote the same like in A and B, but with Euclidean distances.

A FASTING + DASH

V1

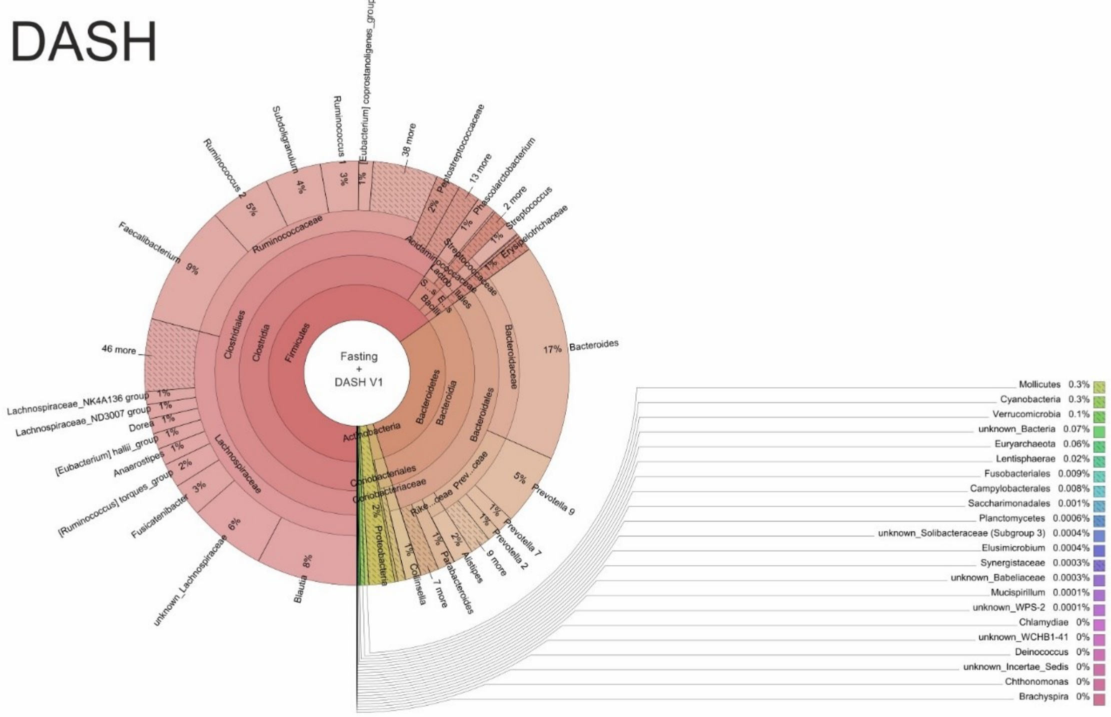

V2

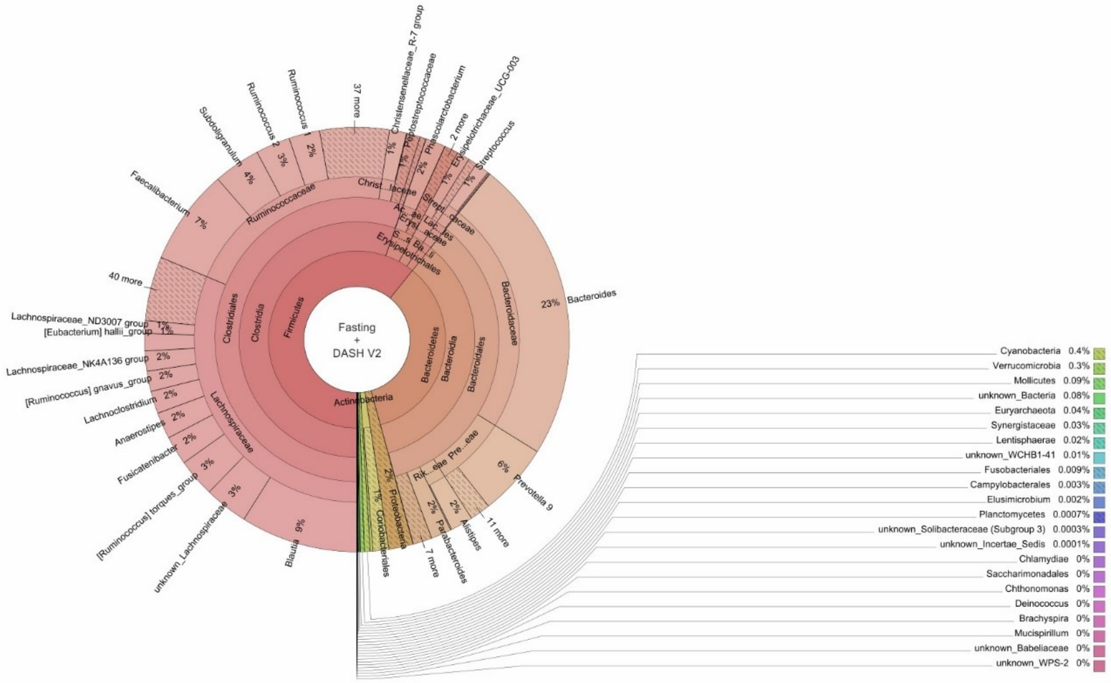

V3

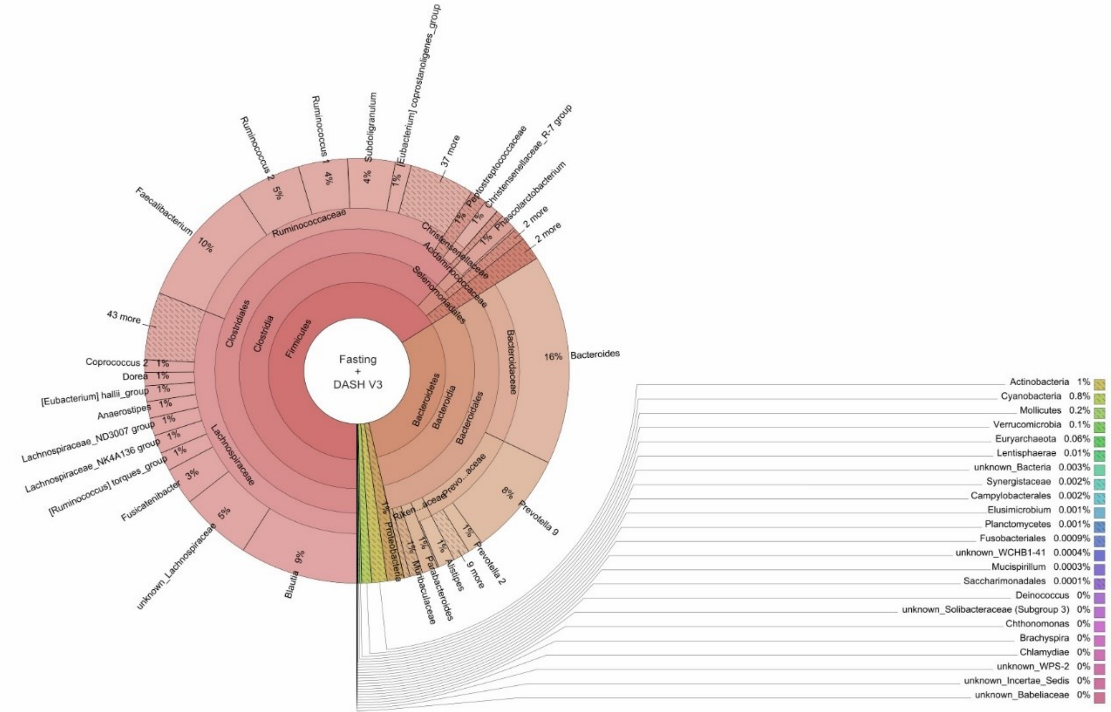

B DASH

V1

V2

V3

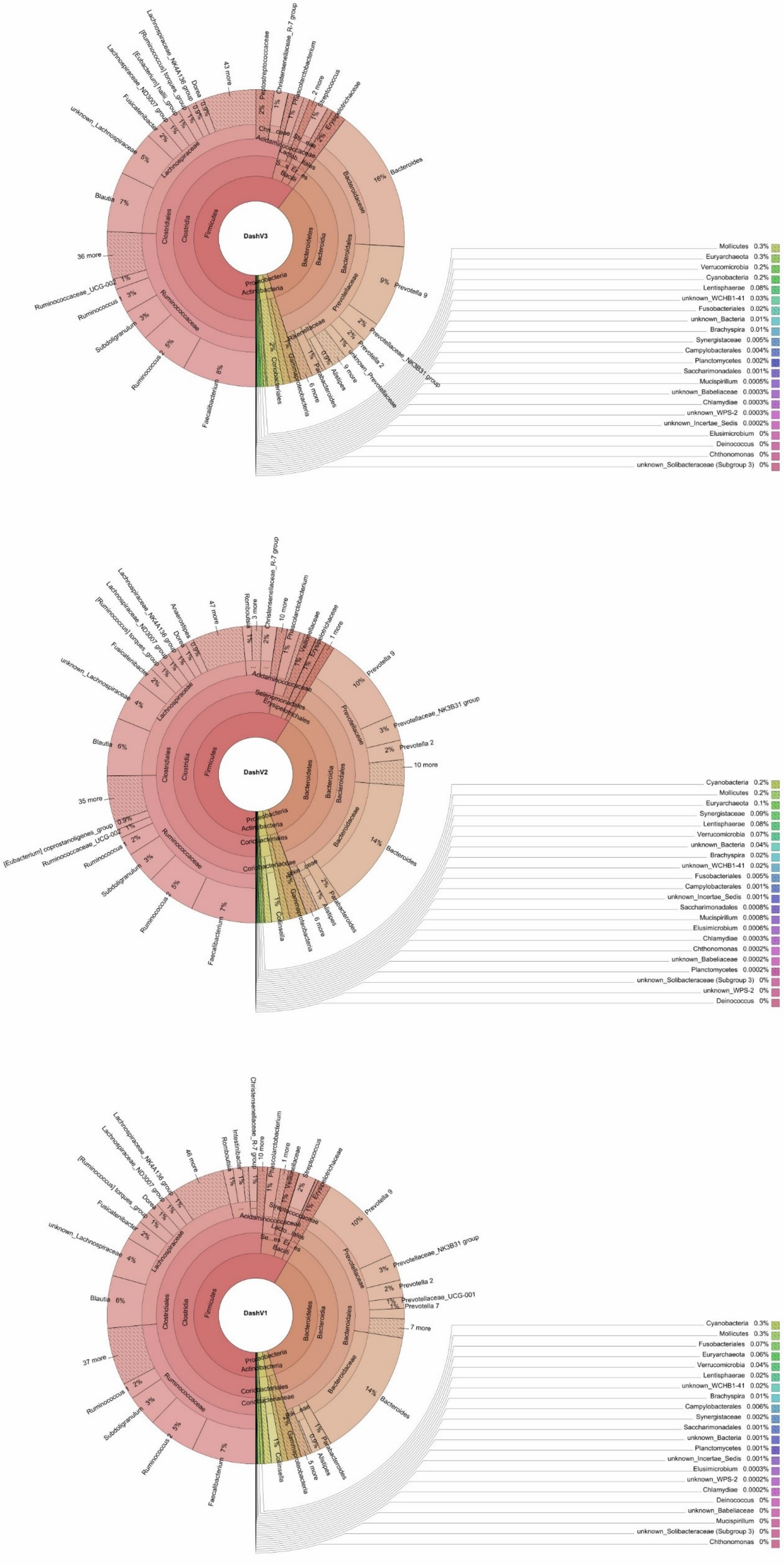

Supplementary Fig. 2. Krona plots of abundances of microbiome taxonomic units derived from 16S stool sequencing. Graphical overview of relative taxonomic composition of gut microbiomes in the study. Stool samples were characterized using 16S sequencing from samples collected at baseline (V1), post fasting / one week of DASH (V2) and after three months (V3) intervention and. (A) Fasting + DASH. (B) DASH.

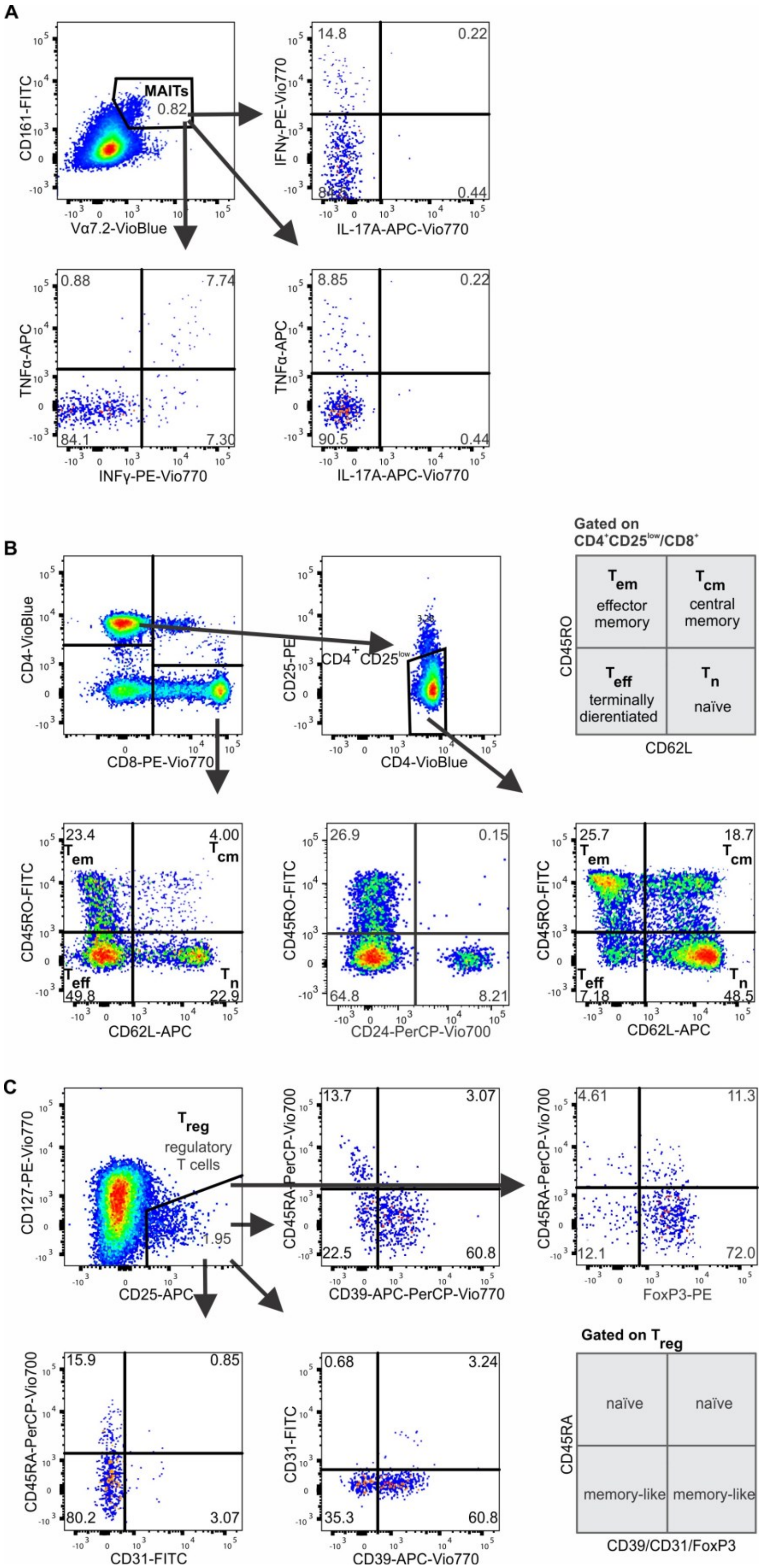
A
MAITs
0.82
CD161-FITC
Vα7.2-VioBlue
IFNγ-PE-Vio770
IL-17A-APC-Vio770
14.8
0.22
0.44
TNFα-APC
INFγ-PE-Vio770
0.88
7.74
84.1
7.30
8.85
0.22
90.5
0.44
B
Gated on CD4+CD25low/CD8+
Tem effector memory
Tcm central memory
Teff terminally dierentiated
Tn naïve
CD45RO
CD62L
CD4-VioBlue
CD8-PE-Vio770
CD25-PE
CD4+ CD25low
CD45RO-FITC
CD62L-APC
CD24-PerCP-Vio700
23.4
4.00
49.8
22.9
26.9
0.15
64.8
8.21
25.7
18.7
7.18
48.5
C
Treg regulatory T cells
1.95
CD127-PE-Vio770
CD25-APC
CD45RA-PerCP-Vio700
CD39-APC-PerCP-Vio770
FoxP3-PE
13.7
3.07
22.5
60.8
4.61
11.3
12.1
72.0
Gated on Treg
naïve
memory-like
CD45RA
CD39/CD31/FoxP3
CD31-FITC
CD39-APC-Vio770
15.9
0.85
80.2
3.07
0.68
3.24
35.3
60.8

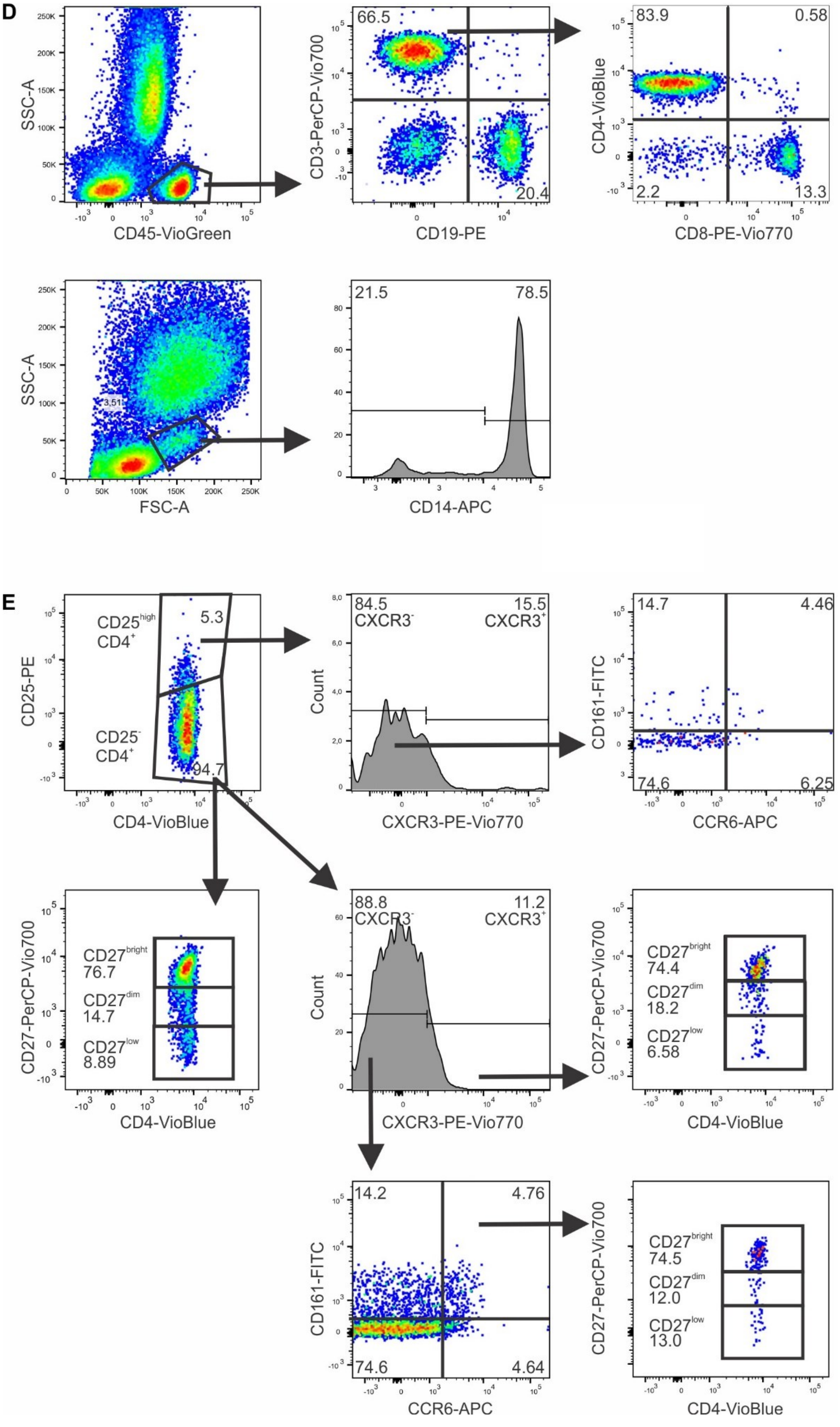
D
SSC-A
CD45-VioGreen
CD3-PerCP-Vio700
CD19-PE
66.5
20.4
CD4-VioBlue
CD8-PE-Vio770
83.9
0.58
2.2
13.3
SSC-A
FSC-A
3.51
CD14-APC
21.5
78.5
E
CD25-PE
CD4-VioBlue
CD25high CD4+
5.3
CD25- CD4+
94.7
Count
CXCR3-PE-Vio770
84.5 CXCR3-
15.5 CXCR3+
CD161-FITC
CCR6-APC
14.7
4.46
74.6
6.25
CD27-PerCP-Vio700
CD27bright 76.7
CD27dim 14.7
CD27low 8.89
88.8 CXCR3-
11.2 CXCR3+
CD27bright 74.4
CD27dim 18.2
CD27low 6.58
14.2
4.76
74.6
4.64
CD27bright 74.5
CD27dim 12.0
CD27low 13.0

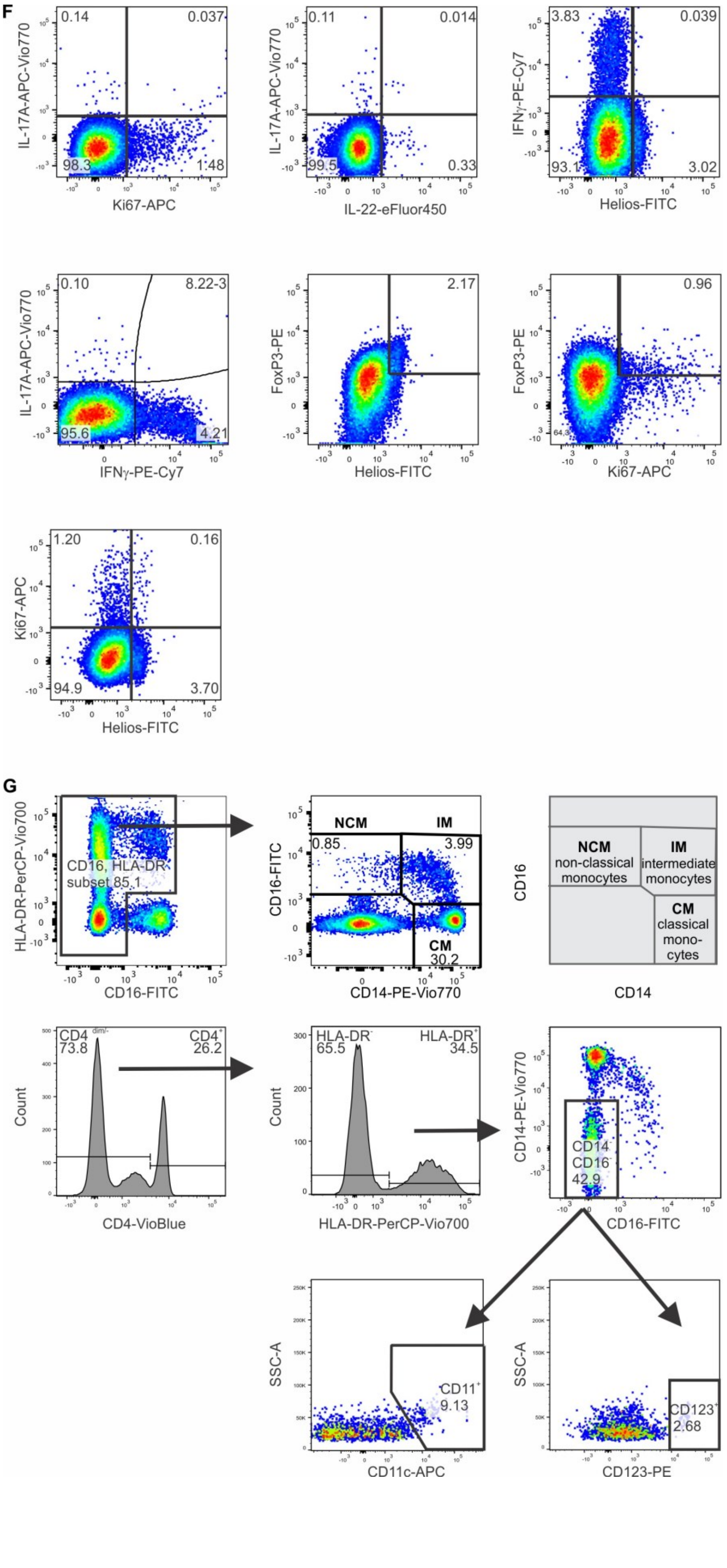
F
IL-17A-APC-Vio770
Ki67-APC
0.14
0.037
98.3
1.48
IL-22-eFluor450
0.11
0.014
99.5
0.33
IFNγ-PE-Cy7
Helios-FITC
3.83
0.039
93.1
3.02
0.10
8.22-3
95.6
4.21
FoxP3-PE
2.17
0.96
64.3
Ki67-APC
1.20
0.16
94.9
3.70
G
HLA-DR-PerCP-Vio700
CD16-FITC
CD16, HLA-DR subset 85.1
CD14-PE-Vio770
NCM
IM
0.85
3.99
CM
30.2
NCM non-classical monocytes
IM intermediate monocytes
CM classical mono-cytes
CD16
CD14
Count
CD4-VioBlue
CD4 dim/- 73.8
CD4+ 26.2
HLA-DR-PerCP-Vio700
HLA-DR- 65.5
HLA-DR+ 34.5
CD14-PE-Vio770
CD14- CD16- 42.9
SSC-A
CD11c-APC
CD11+ 9.13
CD123-PE
CD123+ 2.68

Supplementary Fig. 3. Gating strategies. Events are shown after gating onto FSC-Area vs. SSC-Area, followed by gating on doublet and dead cell exclusion. (A) Mucosa associated invariant T (MAIT) cells were detected in PBMCs depleted of $CD4^+$ cells using magnetic microbead sorting. MAITs were defined as $CD161^+TCRV\alpha 7.2^+CD4\text{-}CD3^+$. After restimulation with phorbol 12-myristate 13-acetate (PMA) and ionomycin for 4hrs, MAITs were stained for IL-17A, TNFα, and IFNγ. (B) Plots show cells gated on live events using LIVE/DEAD Fixable Aqua Dead Cell Stain kit, for 405nm. T cell subsets were gated as $CD8^+CD4^-$, $CD25^-CD4^+CD8^-$, and $CD25^{high}CD4^+CD8^-$. Activation of the T cells was assessed by expression patterns of CD45RO and CD62L. Cells were defined as i) effector memory (Tem): $CD45RO^+CD62L^-$, ii) central memory (Tcm): $CD45RO^+CD62L^+$, iii) naïve (Tn): $CD45RO^-CD62L^+$, iv) terminally differentiated (Teff): $CD45RO^-CD62L^-$, as shown in the scheme to the right. (C) Regulatory T cells (Treg) were defined as $CD4^+CD25^{high}CD127^{low}$ population in isolated PBMCs. Activation status of the cells was detected by the means of surface markers (CD45RA, CD39, and CD31). Representative plots shown. (D) Identification of major cell population from whole blood staining. Whole blood was stained with the respective antibodies followed by the subsequent lysis of the erythrocytes. Samples were measured in a volumetric manner. (E) Identification of Th17 cells and Th17-like $CD25^{high}$ $CD4^+$ cells by surface staining. (F) Intracellular marker expression of $CD4^+$ T cells. $CD4^+$ cells were enriched from PBMCs using magnetic microbead sorting. After restimulation with phorbol 12-myristate 13-acetate (PMA) and ionomycin for 4hrs, cells were stained with the respective antibodies. For the analysis cells were further gated on the $CD3^+$ population. (G) Monocytes were detected from whole peripheral blood mononuclear cell (PBMC) fraction. Monocytes were gated in an $HLA\text{-}DR^+$ or $HLA\text{-}DR^-CD16^-$ subset. Classical, non-classical, and intermediate monocytes were defined as $CD14^{high}CD16^-$, $CD14^{low}CD16^{++}$, and $CD14^+CD16^+$, respectively. Plasmacytoid dendritic cells were defined as $HLA\text{-}DR^+$, $CD14^-$, $CD16^-$, $CD123^+$. Myeloid dendritic cells were defined as $HLA\text{-}DR^+$, $CD14^-$, $CD16^-$, $CD11c^+$. (A-G) correspond to Fig. 1,3,4, (A, E-G) correspond to Fig. 5, (A,F,G) correspond to Fig. 6, (A,B,C,E) correspond to Fig. 7.

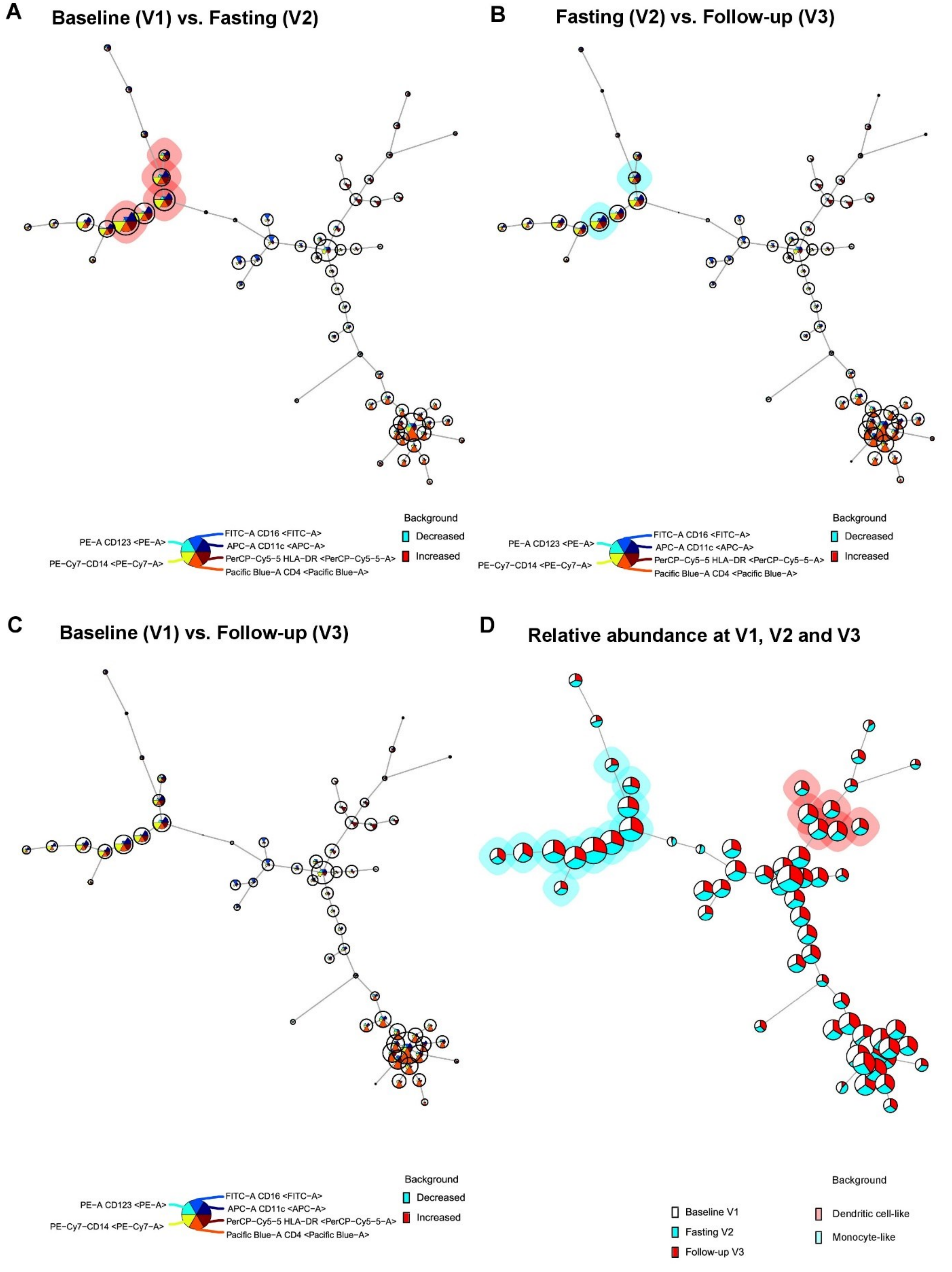

A
Baseline (V1) vs. Fasting (V2)
B
Fasting (V2) vs. Follow-up (V3)
C
Baseline (V1) vs. Follow-up (V3)
D
Relative abundance at V1, V2 and V3
PE-A CD123 <PE-A>
PE-Cy7-CD14 <PE-Cy7-A>
FITC-A CD16 <FITC-A>
APC-A CD11c <APC-A>
PerCP-Cy5-5 HLA-DR <PerCP-Cy5-5-A>
Pacific Blue-A CD4 <Pacific Blue-A>
Background
Decreased
Increased
Baseline V1
Fasting V2
Follow-up V3
Dendritic cell-like
Monocyte-like

Supplementary Fig. 4. FlowSOM analysis of monocytes and antigen-presenting cells (APCs) in the fasting arm. Peripheral blood mononuclear cells were labelled with fluorophore conjugated monoclonal antibodies and measured with multicolor flow cytometry. Data was extracted from the viable gate after doublet-exclusion and down sampled to 6000 events. Samples lacking 6000 events from the viable gate were excluded from the analysis. Monocyte like and APC-like nodules were identified and annotated using the CD markers of the respective node. A-C, Increase (red) or decrease (blue) for Fasting effect (A), Refeeding effect (B), and Study effect (C) is shown within the nodes. D, Quantification of the relative changes between all three time points is shown by the pie chart within the nodes. White, blue and red pie slices refer to V1, V2 and V3, respectively. Blue and green background depicts monocytes and antigen-presenting cells, respectively. (n = 17 for V1, V2 and V3).

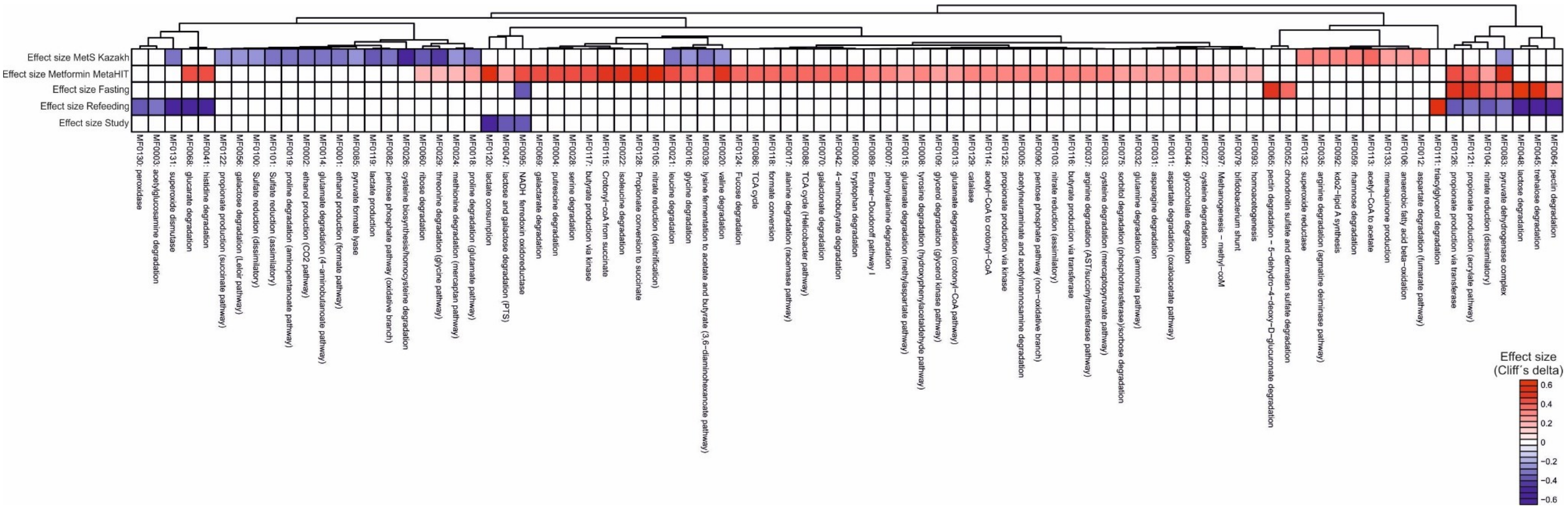

Supplementary Fig. 5. Microbiome functional potential changes during fasting are similar to those induced by metformin and unlike those characteristic for MetS. To compare the microbiome signatures of fasting and refeeding to those seen in metabolic syndrome and in the treatment of insulin resistance with metformin, we reanalyzed gut microbiome data from two previous studies powered to test these two factors, respectively. To test associations between gut microbiome functional module (GMM) abundances and metabolic syndrome status, we reanalyzed samples from Kushgulova et al.[1], controlling for metformin treatment. To test associations between gut microbiome functional module (GMM) abundances and metformin treatment, we reanalyzed samples from Forslund et al.[2], controlling for metabolic syndrome status. Size and direction (Cliff's delta) of significant effects are shown in the heatmap alongside corresponding signals from the present study. Fasting, recovery, and overall study effect from our novel data are shown. Blue and red indicate significant (MWU FDR < 0.1 for metformin/MetS status, respectively, post-hoc nested model test for confounder (the other variable) P < 0.05) depletion/enrichment in each data set. White indicates non-significant effect or absence of the module in a dataset. Modules significantly different in abundance in the metformin substudy show some overlap with and similar directional changes as in our fasting study, whereas recovery exhibits the opposing pattern. MetS and metformin functional signals are starkly different from one another, however there is little overlap between features altered in MetS and by fasting in our novel data.

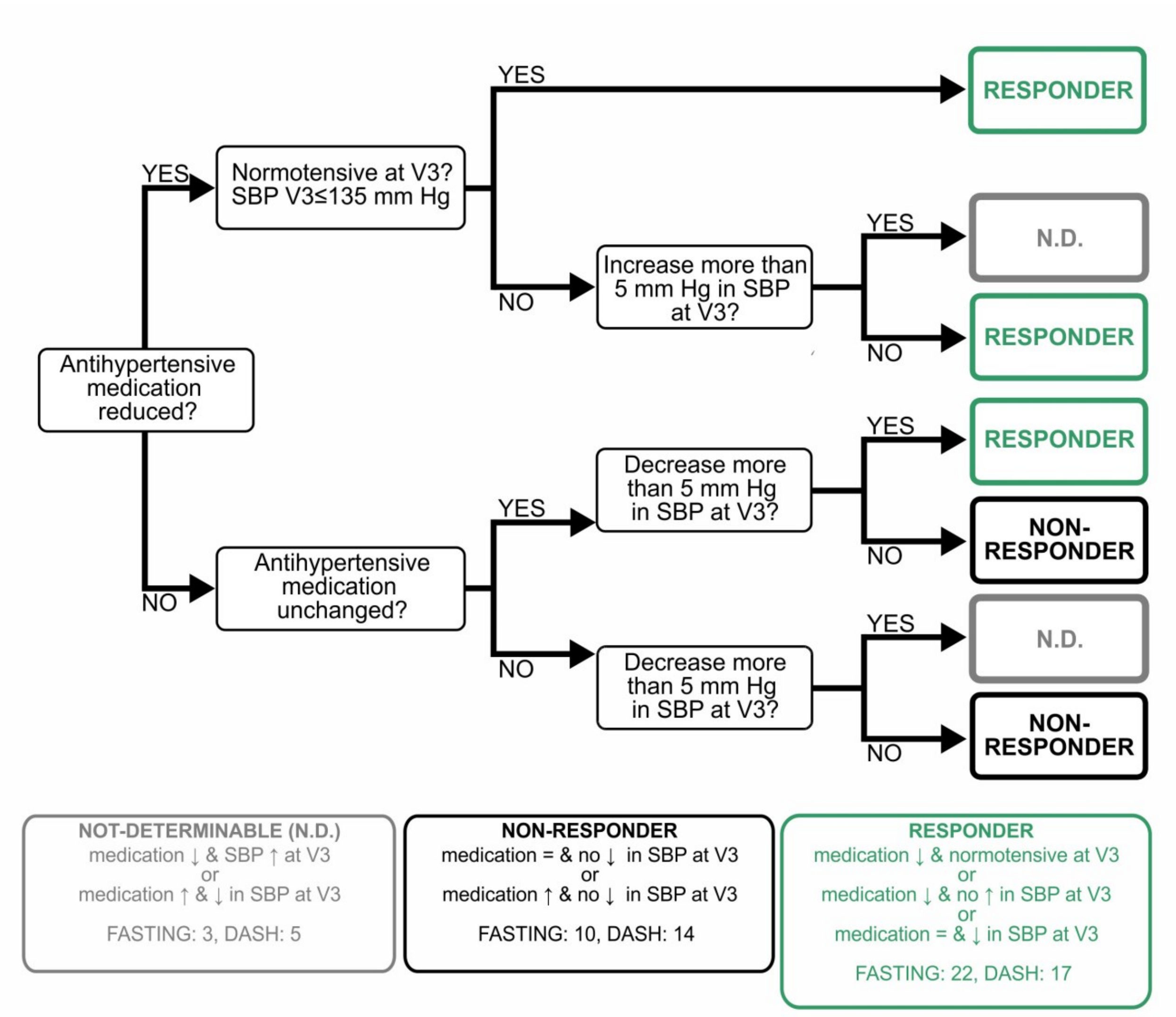

Supplementary Fig. 6. Decision tree for the determination of blood-pressure responders and non-responders. Individual 24h systolic ambulatory blood pressure (SBP) and individual anti-hypertensive medication at 3 months (V3) was evaluated in relation to baseline (V1) and patients were categorized as responder, non-responder or not determinable. Normotensive SBP was defined as being ≤ 135 mm Hg. Significant change in the SBP was defined as an increase or decrease being ≥ 5 mmHg. Patients were categorized as responders if at V3: i) their anti-hypertensive medication was reduced and SBP was ≤ 135 mm Hg, ii) anti-hypertensive medication was reduced and SBP did not increase significantly, iii) if their anti-hypertensive medication did not change and their SBP significantly decreased (Fasting n=22, DASH n= 17). Patients were categorized as non-responders if at V3: i) their anti-hypertensive medication was not changed and SBP did not decrease significantly, ii) if their anti-hypertensive medication was increased but SBP did not decrease (Fasting n= 10, DASH n = 14). Patients were categorized as not-determinable if at V3: i) their anti-hypertensive medication was decreased and SBP significantly increased, ii) their anti-hypertensive medication was increased and SBP significantly decreased (Fasting n = 3, DASH = 5).

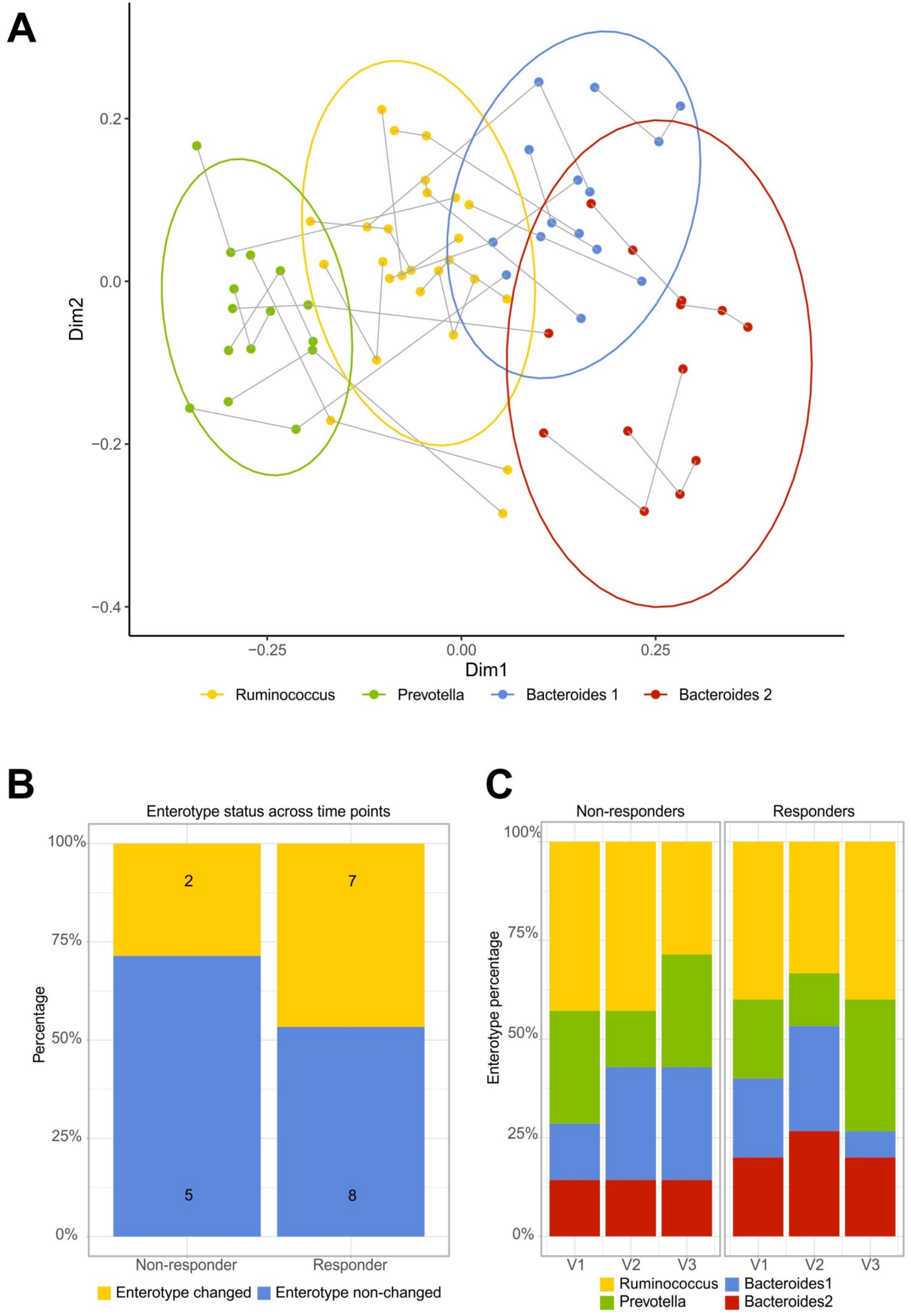
A
Dim2
Dim1
0.2
0.0
−0.2
−0.4
−0.25
0.00
0.25
Ruminococcus
Prevotella
Bacteroides 1
Bacteroides 2
B
Enterotype status across time points
Percentage
100%
75%
50%
25%
0%
2
7
5
8
Non-responder
Responder
Enterotype changed
Enterotype non-changed
C
Non-responders
Responders
Enterotype percentage
V1
V2
V3
Ruminococcus
Bacteroides1
Prevotella
Bacteroides2

Supplementary Fig. 7. Enterotype of fasting arm samples. Four enterotypes were identified, which we classify as *Ruminococcus*-dominated, *Prevotella*-dominated, and two *Bacteroides*-dominated enterotypes. (A) Fecal microbiome (n = 69) community variation on genus level, shown in a principal coordinates analysis based on Bray-Curtis dissimilarities. Four enterotypes are resolved, and shown using different colors. Paired samples are linked by grey lines. Ellipses indicate 95% confidence interval. (B) Percentage of individuals with changed or unchanged enterotype throughout the three time points (pre-intervention, post-fasting, followup). Raw counts of samples falling within each outcome group are shown in the bars. (C) Proportion of enterotypes in each case represented by stacked bar plot across time points. Most individuals retained the same enterotype throughout the intervention, with a trend of change in enterotype (chi-squared test, P = 0.65).

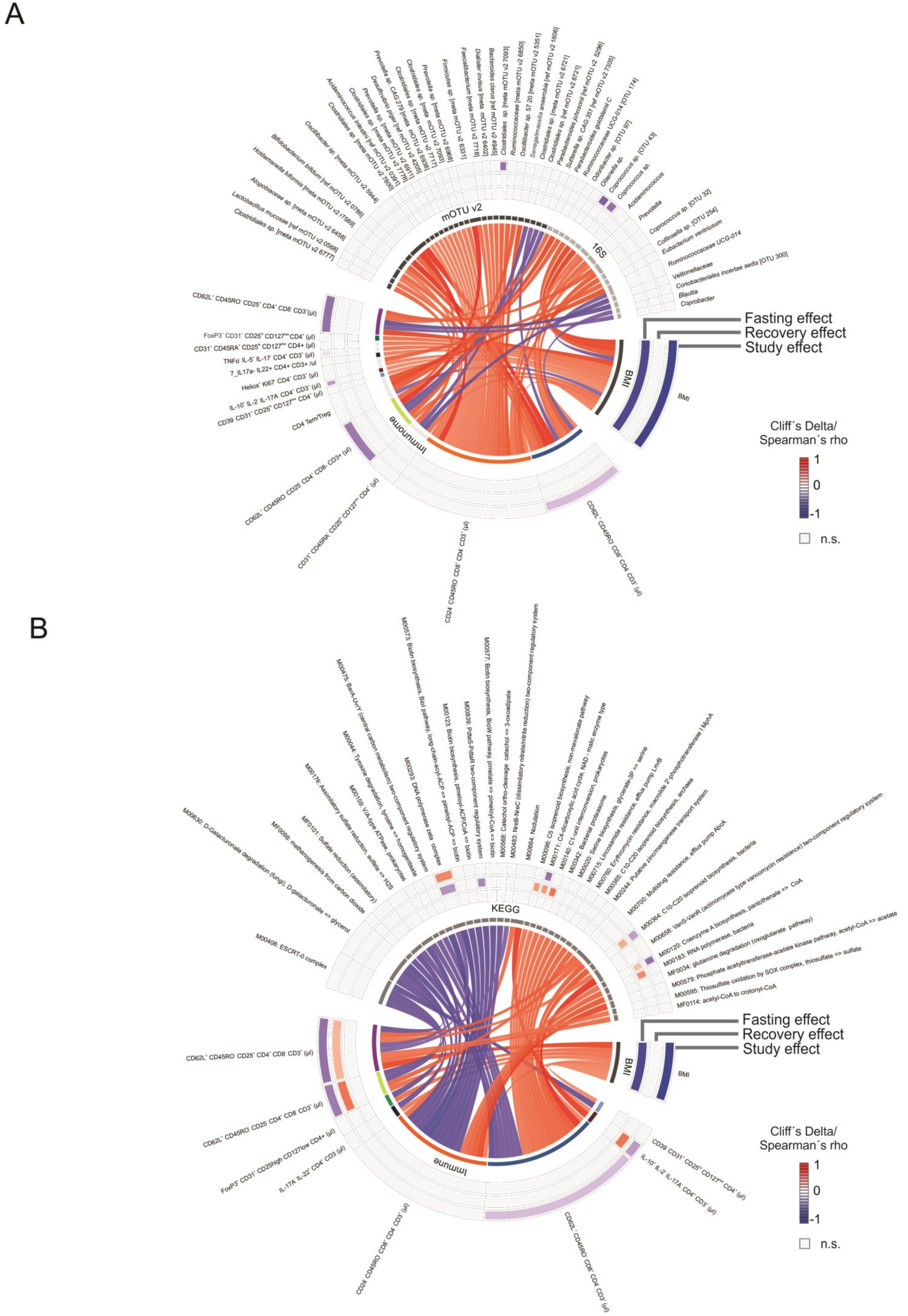

A
mOTU v2
16S
Immunome
BMI
Fasting effect
Recovery effect
Study effect
Cliff´s Delta/
Spearman´s rho
1
0
-1
n.s.
Clostridiales sp. [meta mOTU v2 6777]
Lactobacillus mucosae [ref mOTU v2 0568]
Atopobiaceae sp. [meta mOTU v2 6458]
Holdemanella biformis [meta mOTU v2 r7589]
Bifidobacterium bifidum [ref mOTU v2 0786]
Oscillibacter sp. [meta mOTU v2 5944]
Clostridiales sp. [meta mOTU v2 7800]
Acidaminococcus intestini [ref mOTU v2 0391]
Prevotella sp. [meta mOTU v2 7778]
Desulfovibrio piger [ref mOTU v2 6911]
Prevotella sp. CAG:279 [meta mOTU v2 4205]
Clostridiales sp. [meta mOTU v2 6936]
Clostridiales sp. [meta mOTU v2 7711]
Prevotella sp. [meta mOTU v2 7095]
Firmicutes sp. [meta mOTU v2 6968]
Faecalibacterium [meta mOTU v2 6331]
Dialister invisus [meta mOTU v2 7718]
Bacteroides clarus [ref mOTU v2 6402]
Clostridiales sp. [meta mOTU v2 4945]
Ruminococcaceae [meta mOTU v2 7093]
Oscillibacter sp. 57 20 [meta mOTU v2 6850]
Senegalimassilia anaerobia [ref mOTU v2 5351]
Clostridiales sp. [meta mOTU v2 1696]
Parabacteroides johnsonii [ref mOTU v2 6721]
Sutterella sp. CAG:351 [ref mOTU v2 6721]
Parabacteroides goldsteinii C
Ruminococcaceae UCG-014 [OTU 174]
Odoribacter sp. [OTU 97]
Olsenella sp.
Coprococcus sp. [OTU 43]
Coprococcus sp.
Acidaminococcus
Prevotella
Coprococcus sp. [OTU 32]
Collinsella sp. [OTU 254]
Eubacterium ventriosum
Ruminococcaceae UCG-014
Veillonellaceae
Coriobacteriales incertae sedis [OTU 300]
Blautia
Coprobacter
BMI
CD62L⁺ CD45RO⁻ CD25⁺ CD4⁺ CD8⁻ CD3⁺(µl)
FoxP3⁺ CD31⁺ CD25ʰⁱ CD127ˡᵒʷ CD4⁺ (µl)
CD31⁺ CD45RA⁺ CD25ʰⁱ CD127ˡᵒʷ CD4+ (µl)
TNFα⁻ IL-5⁺ IL-17⁻ CD4⁺ CD3⁺ (µl)
7_IL17a- IL22+ CD4+ CD3+ /ul
Helios⁺ Ki67⁻ CD4⁺ CD3⁺ (µl)
IL-10⁺ IL-2⁻ IL-17A⁻ CD4⁺ CD3⁺ (µl)
CD39⁻ CD31⁺ CD25ʰⁱ CD127ˡᵒʷ CD4⁺ (µl)
CD4 Tem/Treg
CD62L⁺ CD45RO⁻ CD25⁻ CD4⁺ CD8- CD3+ (µl)
CD31⁺ CD45RA⁻ CD25⁺ CD127ˡᵒʷ CD4⁺ (µl)
CD24⁻ CD45RO⁻ CD8⁺ CD4⁻ CD3⁺ (µl)
CD62L⁻ CD45RO⁻ CD8⁺ CD4⁻ CD3⁺ (µl)
B
KEGG
Immune
BMI
Fasting effect
Recovery effect
Study effect
Cliff´s Delta/
Spearman´s rho
1
0
-1
n.s.
M00408: ESCRT-0 complex
M00630: D-Galacturonate degradation (fungi), D-galacturonate => glycerol
MF0098: methanogenesis from carbon dioxide
MF0101: Sulfate reduction (assimilatory)
M00176: Assimilatory sulfate reduction, sulfate => H2S
M00159: V/A-type ATPase, prokaryotes
M00044: Tyrosine degradation, tyrosine => homogentisate
M00475: BarA-UvrY (central carbon metabolism) two-component regulatory system
M00293: DNA polymerase zeta complex
M00573: Biotin biosynthesis, BioI pathway, long-chain-acyl-ACP => pimeloyl-ACP => biotin
M00123: Biotin biosynthesis, pimeloyl-ACP/CoA => biotin
M00839: PdtaS-PdtaR two-component regulatory system
M00577: Biotin biosynthesis, BioW pathway, pimelate => pimeloyl-CoA => biotin
M00568: Catechol ortho-cleavage, catechol => 3-oxoadipate
M00483: NreB-NreC (dissimilatory nitrate/nitrite reduction) two-component regulatory system
M00664: Nodulation
M00096: C5 isoprenoid biosynthesis, non-mevalonate pathway
M00171: C4-dicarboxylic acid cycle, NAD - malic enzyme type
M00140: C1-unit interconversion, prokaryotes
M00342: Bacterial proteasome
M00020: Serine biosynthesis, glycerate-3P => serine
M00715: Lincosamide resistance, efflux pump LmrB
M00780: Erythromycin resistance, macrolide 2'-phosphotransferase I MphA
M00365: C10-C20 isoprenoid biosynthesis, archaea
M00244: Putative zinc/manganese transport system
M00700: Multidrug resistance, efflux pump AbcA
M00364: C10-C20 isoprenoid biosynthesis, bacteria
M00658: VanS-VanR (actinomycete type vancomycin resistance) two-component regulatory system
M00120: Coenzyme A biosynthesis, pantothenate => CoA
M00183: RNA polymerase, bacteria
MF0034: glutamine degradation (oxoglutarate pathway)
M00579: Phosphate acetyltransferase-acetate kinase pathway, acetyl-CoA => acetate
M00595: Thiosulfate oxidation by SOX complex, thiosulfate => sulfate
MF0114: acetyl-CoA to crotonyl-CoA
BMI
CD62L⁺ CD45RO⁻ CD25⁺ CD4⁺ CD8⁻ CD3⁺ (µl)
CD62L⁺ CD45RO⁻ CD25 CD4⁺ CD8⁻ CD3⁺ (µl)
FoxP3⁺ CD31⁺ CD25high CD127low CD4+ (µl)
IL-17A⁻ IL-22⁺ CD4⁺ CD3 (µl)
CD24 CD45RO CD8⁺ CD4 CD3⁺ (µl)
CD62L⁻ CD45RO CD8⁺ CD4 CD3⁺ (µl)
IL-10⁺ IL-2⁻ IL-17A⁻ CD4⁺ CD3⁺ (µl)
CD39⁻ CD31⁺ CD25ʰⁱ CD127ˡᵒʷ CD4⁺ (µl)

Supplementary Fig. 8. Association between the immunome, microbiome features and BMI. Chord diagrams visualize the interrelation between body mass index (BMI), fasting impacted microbiome functional or taxonomic features, and immune cell subsets. Features are shown that form triplets of immune, microbial and phenotype variables where at least two of three correlations are significant (Spearman FDR < 0.05, post-hoc nested model test accounting for same-donor samples < 0.05) in the fasting arm of our cohort, and where in addition one or more feature significantly (drug-adjusted post-hoc FDR < 0.05) are affected by the intervention. Color of the connectors indicates positive or negative correlation (Spearman´s rho), color of the cells within the tracks indicates changes upon fasting, refeeding or the overall study effect (Cliff´s delta). (A) BMI/immune/taxonomic microbiome interrelations, (B) BMI/immune/microbiome KEGG annotation interrelations. Identical non-gray colors of the inner track of immune features denote the same immune features on (A) and (B).

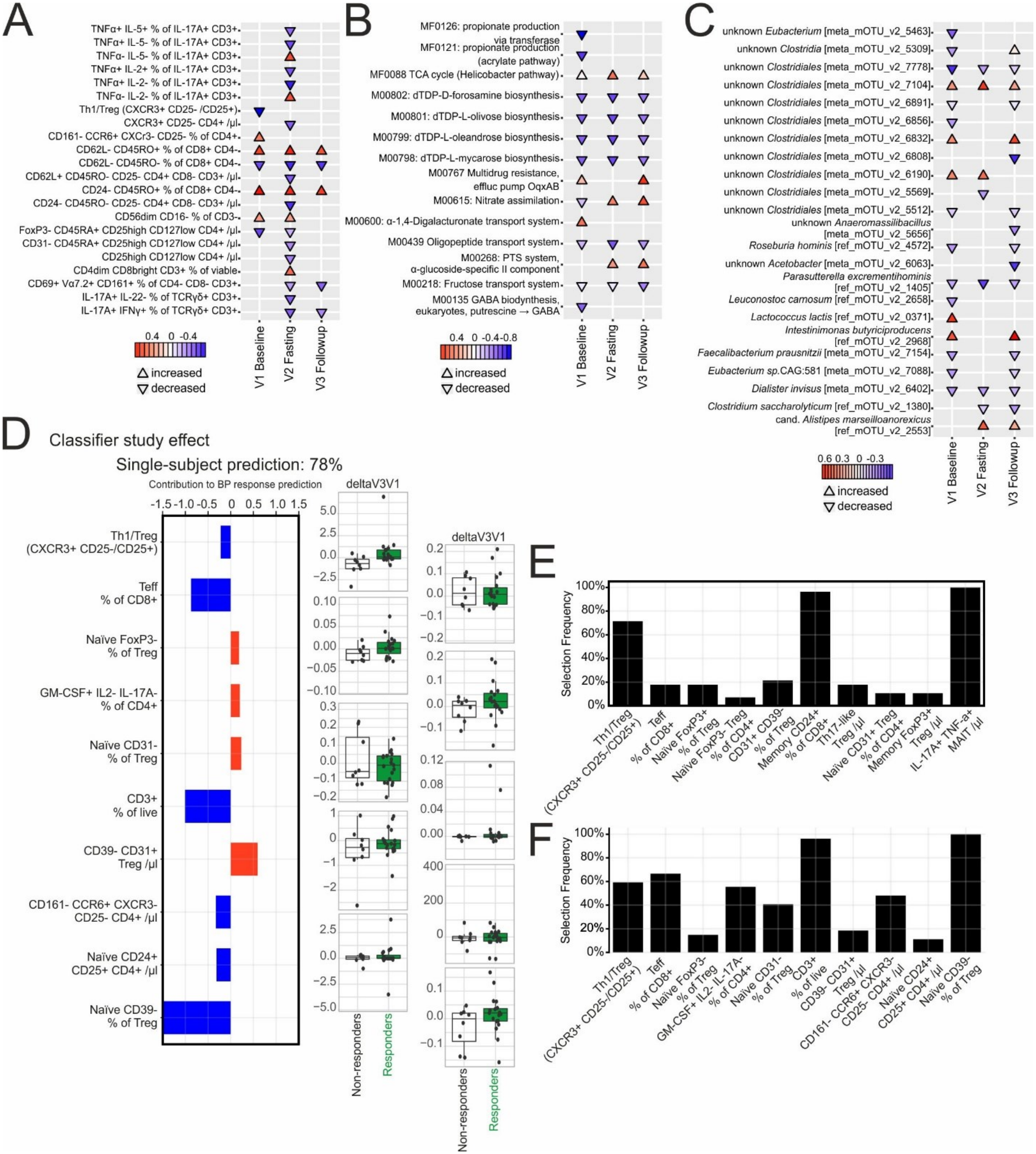
A
B
C
D
E
F
Classifier study effect
Single-subject prediction: 78%
Contribution to BP response prediction
deltaV3V1
V1 Baseline
V2 Fasting
V3 Followup
increased
decreased
Non-responders
Responders
Selection Frequency

Supplementary Fig. 9. Long-lasting blood pressure responders and non-responders differ in microbiome and immunome composition. (A-C) Cuneiform plot shows effect sizes (Cliff's delta; hue and marker size shows effect size, marker direction shows sign of effect) of immunome features (A), gut functional profiles using KEGG and GMM (B), and gut taxon abundances assessed using the mOTUv2 framework (C) significantly (posthoc FDR < 0.05) differing between responders and non-responders at the different time points. (D), Prediction model for blood pressure response using the changes of immune features between baseline (V1) and follow-up (V3). Single subject prediction was quantified using a leave-one-out cross-validation approach. Ten immune cell features were used to build up a multivariate logistic-regression algorithm. The bar plots represent the regression in a model with binary output (responder yes=1 vs no=0) for every feature. (E), Selection frequency of the different parameters for the prediction model shown in Fig. 4c over the different classifiers built by using a leave-one-out cross-validation. (F), Selection frequency of the different parameters for the prediction model shown in (D) over the different classifiers built by using a leave-one-out cross-validation. Treg: $CD25^{high}CD127^{low}CD4^{+}$.

Supplementary References

1. Kushugulova A*, et al.* Metagenomic analysis of gut microbial communities from a Central Asian population. *BMJ Open* **8**, e021682 (2018).

2. Forslund K*, et al.* Disentangling type 2 diabetes and metformin treatment signatures in the human gut microbiota. *Nature* **528**, 262-266 (2015).

3. Frost F*, et al.* A structured weight loss program increases gut microbiota phylogenetic diversity and reduces levels of Collinsella in obese type 2 diabetics: A pilot study. *PLoS One* **14**, e0219489 (2019).

4. Ozkul C, Yalinay M, Karakan T. Structural changes in gut microbiome after Ramadan fasting: a pilot study. *Beneficial microbes* **11**, 227-233 (2020).

5. Louis S, Tappu RM, Damms-Machado A, Huson DH, Bischoff SC. Characterization of the Gut Microbial Community of Obese Patients Following a Weight-Loss Intervention Using Whole Metagenome Shotgun Sequencing. *PLoS One* **11**, e0149564 (2016).

6. Velikonja A, Lipoglavsek L, Zorec M, Orel R, Avgustin G. Alterations in gut microbiota composition and metabolic parameters after dietary intervention with barley beta glucans in patients with high risk for metabolic syndrome development. *Anaerobe* **55**, 67-77 (2019).

7. Roager HM*, et al.* Whole grain-rich diet reduces body weight and systemic low-grade inflammation without inducing major changes of the gut microbiome: a randomised cross-over trial. *Gut* **68**, 83-93 (2019).

8. Liu Z*, et al.* Gut microbiota mediates intermittent-fasting alleviation of diabetes-induced cognitive impairment. *Nat Commun* **11**, 855 (2020).

9. Kopf JC*, et al.* Role of whole grains versus fruits and vegetables in reducing subclinical inflammation and promoting gastrointestinal health in individuals affected by overweight and obesity: a randomized controlled trial. *Nutrition journal* **17**, 72 (2018).

10. Guevara-Cruz M*, et al.* Improvement of Lipoprotein Profile and Metabolic Endotoxemia by a Lifestyle Intervention That Modifies the Gut Microbiota in Subjects With Metabolic Syndrome. *Journal of the American Heart Association* **8**, e012401 (2019).
