# Supplementary material for: Fasting alters the gut microbiome reducing blood pressure and body weight in metabolic syndrome patients
Source: Nat Commun. 2021 Mar 30;12:1970. doi: 10.1038/s41467-021-22097-0 (PMC8010079; doi:10.1038/s41467-021-22097-0)
Supplement: Supplementary file 3 — Description of Additional Supplementary Files [file 41467_2021_22097_MOESM3_ESM.pdf]

Description of Additional Supplementary Files

Supplementary Data 1
Alpha diversities of the microbiome data.

Supplementary Data 2
Univariate analysis of the fasting+DASH group.

Supplementary Data 3
Drug data.

Supplementary Data 4
Blood pressure responsiveness.

Supplementary Data 5
BMI adjusted univariate analysis.

Supplementary Data 6
Comparison of clinical characteristics of BP responders and non-responders at baseline of the fasting+DASH arm.

Supplementary Data 7
Univariate analysis of the Responders in the fasting+DASH arm.

Supplementary Data 8
Univariate analysis of the DASH arm.

Supplementary Data 9
Triangulation analysis.

Supplementary Data 10
References for poorly characterized SCFA-producing microbes.

Supplementary Data 11
Re-analysis of the Mesnage data.

Supplementary Data 12
Classification of the volunteers of the Mesnage dataset according to their BP decrease after three months. A $\geq$ 5 mmHg decrease of the SBP at three months compared to baseline was considered to be significant.

Supplementary Data 13
Review of the studies related investigating the relation of dietary interventions, gut microbiome and metabolic diseases, including (cardiovascular) health, MetS, diabetes, obesity.

Supplementary Data 14
Mapping statistics for human contamination, IGC, refMG and mOTUsv2 using ngless and bwa.
