# Supplementary material for: Fasting alters the gut microbiome reducing blood pressure and body weight in metabolic syndrome patients
Source: Nat Commun. 2021 Mar 30;12:1970. doi: 10.1038/s41467-021-22097-0 (PMC8010079; doi:10.1038/s41467-021-22097-0)
Supplement: Supplementary file 5 — Supplementary Data 2 [file 41467_2021_22097_MOESM5_ESM.pdf]

Supplementary Data 2 Alpha diversities

Fasting+ DASH

Feature	Space	Fasting effect	Recovery effect	Study effect	FDR (intervention model comparison)	Fasting effect size (Cliff's delta)	Fasting FDR (MWU post-hoc, N=3)	Recovery effect size (Cliff's delta)	Recovery FDR (MWU post-hoc, N=3)	Study effect size (Cliff's delta)	Study FDR (MWU post-hoc, N=3)	Signal
Shannon	Microbiome-Diversity	NS	NS	NS	0.873	-0.217	0.269	0.217	0.209	-0.043	0.870	INSIGNIFICANT
InverseSimpson	Microbiome-Diversity	NS	NS	NS	0.570	-0.391	0.147	0.391	0.147	-0.130	0.360	INSIGNIFICANT
Richness	Microbiome-Diversity	NS	NS	NS	0.535	-0.391	0.604	-0.043	0.651	-0.043	0.604	INSIGNIFICANT
Chao1	Microbiome-Diversity	NS	NS	NS	0.449	-0.130	0.501	-0.043	0.501	-0.217	0.402	INSIGNIFICANT
Simpson	Microbiome-Diversity	NS	NS	NS	0.570	-0.391	0.098	0.391	0.098	-0.130	0.482	INSIGNIFICANT
Eveness	Microbiome-Diversity	NS	NS	NS	0.570	-0.130	0.240	0.478	0.054	0.130	0.917	INSIGNIFICANT

Fasting+DASH responders only

Feature	Space	Fasting effect	Recovery effect	Study effect	FDR (intervention model comparison)	Fasting effect size (Cliff's delta)	Fasting FDR (MWU post-hoc, N=3)	Recovery effect size (Cliff's delta)	Recovery FDR (MWU post-hoc, N=3)	Study effect size (Cliff's delta)	Study FDR (MWU post-hoc, N=3)	Signal
Chao1	Microbiome-Diversity	NS	NS	NS	0.684	0.067	0.890	-0.067	0.842	-0.333	0.623	INSIGNIFICANT
Shannon	Microbiome-Diversity	NS	NS	NS	0.956	-0.200	0.733	0.200	0.563	0.067	0.934	INSIGNIFICANT
InverseSimpson	Microbiome-Diversity	NS	NS	NS	0.684	-0.600	0.124	0.333	0.188	-0.333	0.181	INSIGNIFICANT
Richness	Microbiome-Diversity	NS	NS	NS	0.684	-0.200	0.955	0.000	0.955	0.067	0.955	INSIGNIFICANT
Eveness	Microbiome-Diversity	NS	NS	NS	0.956	-0.200	0.539	0.333	0.284	0.067	0.847	INSIGNIFICANT
Simpson	Microbiome-Diversity	NS	NS	NS	0.684	-0.600	0.181	0.333	0.181	-0.333	0.277	INSIGNIFICANT

DASH only

Feature	Space	Fasting effect	Recovery effect	Study effect	FDR (intervention model comparison)	Fasting effect size (Cliff's delta)	Fasting FDR (MWU post-hoc, N=3)	Recovery effect size (Cliff's delta)	Recovery FDR (MWU post-hoc, N=3)	Study effect size (Cliff's delta)	Study FDR (MWU post-hoc, N=3)	Signal
Richness	Microbiome-Diversity	NS	NS	NS	0.506	-0.043	0.594	0.174	0.518	0.130	0.594	INSIGNIFICANT
InverseSimpson	Microbiome-Diversity	NS	NS	NS	0.136	0.391	0.227	-0.043	0.870	0.130	0.227	INSIGNIFICANT
Simpson	Microbiome-Diversity	NS	NS	NS	0.136	0.391	0.480	-0.043	0.893	0.130	0.840	INSIGNIFICANT
Chao1	Microbiome-Diversity	NS	NS	NS	0.293	-0.130	0.501	0.130	0.480	0.304	0.501	INSIGNIFICANT
Shannon	Microbiome-Diversity	NS	NS	NS	0.148	0.304	0.517	0.043	0.964	0.130	0.517	INSIGNIFICANT
Eveness	Microbiome-Diversity	NS	NS	NS	0.148	0.391	0.494	-0.043	0.665	0.130	0.494	INSIGNIFICANT

Fasting+DASH, responders vs. non-responders

Feature	Space	Baseline	Fasting	Followup	Baseline responder MWU P	Baseline responder FDR	Baseline responder effect size (Cliff's delta)	Fasting responder MWU P	Fasting responder FDR	Fasting responder effect size (Cliff's delta)	Followup responder MWU P	Followup responder FDR	Followup responder effect size (Cliff's delta)
Richness	Microbiome-Diversity	NS	NS	NS	0.724	0.071	-0.105	0.916	0.046	0.038	0.621	0.000	-0.142857143
Simpson	Microbiome-Diversity	NS	NS	NS	0.447	0.650	0.219	0.837	0.907	0.067	0.891	0.000	-0.047619048
Eveness	Microbiome-Diversity	NS	NS	NS	0.581	0.650	0.162	0.490	0.907	0.200	0.731	0.001	-0.104761905
Shannon	Microbiome-Diversity	NS	NS	NS	0.731	0.650	0.105	0.407	0.700	0.238	0.891	0.000	-0.047619048
Chao1	Microbiome-Diversity	NS	NS	NS	1.000	0.212	0.010	0.945	0.046	-0.029	0.731	0.000	-0.104761905
InverseSimpson	Microbiome-Diversity	NS	NS	NS	0.447	0.716	0.219	0.837	0.092	0.067	0.891	0.000	-0.047619048
