# Supplementary material for: Fasting alters the gut microbiome reducing blood pressure and body weight in metabolic syndrome patients
Source: Nat Commun. 2021 Mar 30;12:1970. doi: 10.1038/s41467-021-22097-0 (PMC8010079; doi:10.1038/s41467-021-22097-0)
Supplement: Supplementary file 7 — Supplementary Data 4 [file 41467_2021_22097_MOESM7_ESM.pdf]

Supplementary Data 4

patientID	Group	SBP ABPM 24h Resp
1	Fasting arm	non-R
2	Fasting arm	R
3	Fasting arm	R
4	DASH arm	R
5	DASH arm	non-R
7	DASH arm	non-R
8	DASH arm	non-R
9	DASH arm	not-determinable
11	DASH arm	R
12	Fasting arm	non-R
13	Fasting arm	R
14	DASH arm	R
15	DASH arm	R
16	Fasting arm	R
17	Fasting arm	non-R
18	DASH arm	R
19	DASH arm	R
20	DASH arm	non-R
21	Fasting arm	R
22	Fasting arm	non-R
23	DASH arm	not-determinable
24	Fasting arm	R
25	DASH arm	R
26	Fasting arm	not-determinable
27	DASH arm	R
28	Fasting arm	R
29	DASH arm	non-R
30	Fasting arm	R
31	DASH arm	non-R
32	DASH arm	R
33	Fasting arm	R
34	Fasting arm	not-determinable
35	Fasting arm	R
36	DASH arm	non-R
37	Fasting arm	R
38	DASH arm	non-R
39	Fasting arm	non-R
40	DASH arm	R
41	DASH arm	R
42	DASH arm	R
43	Fasting arm	R
44	DASH arm	non-R
45	Fasting arm	R
46	Fasting arm	non-R
47	Fasting arm	R
48	DASH arm	not-determinable
49	Fasting arm	non-R
50	Fasting arm	R
51	DASH arm	not-determinable
52	Fasting arm	R
53	DASH arm	non-R
54	DASH arm	non-R
55	Fasting arm	non-R
56	DASH arm	R
57	DASH arm	non-R
58	Fasting arm	R
59	Fasting arm	not-determinable
60	DASH arm	R
61	Fasting arm	R
62	Fasting arm	R
63	DASH arm	non-R
64	Fasting arm	R
65	Fasting arm	non-R
66	DASH arm	R
67	Fasting arm	R
68	Fasting arm	R
69	DASH arm	R
70	DASH arm	R
72	DASH arm	not-determinable
73	DASH arm	non-R
74	Fasting arm	non-R

non-R	=non-responder
R	=responder
not-determinable	
