# Supplementary material for: Fasting alters the gut microbiome reducing blood pressure and body weight in metabolic syndrome patients
Source: Nat Commun. 2021 Mar 30;12:1970. doi: 10.1038/s41467-021-22097-0 (PMC8010079; doi:10.1038/s41467-021-22097-0)
Supplement: Supplementary file 9 — Supplementary Data 6 [file 41467_2021_22097_MOESM9_ESM.pdf]

Supplementary Data 6. Comparison of clinical characteristics of BP responders and non-responders at baseline of the fasting+DASH arm.

	Responders median	Non-responders Median	MWU P value	FDR-adjusted P value
Age (year)	57.0	58.5	0.439	0.636
Height (cm)	173.0	169.0	0.199	0.581
Office SBP (mm Hg)	135.8	134.0	0.329	0.584
Office DBP (mm Hg)	87.8	83.8	0.272	0.581
24h ABPM SBP (mm Hg)	135.5	126.7	0.095	0.581
24h ABPM DBP (mm Hg)	81.0	78.5	0.409	0.636
24h ABPM MAP (mm Hg)	106.0	100.5	0.167	0.581
24h ABPM peripheral resistance (mm Hg*s/ml)	1.3	1.2	0.017	0.529
SBP day (mm Hg)	138.0	128.0	0.264	0.581
DBP day (mm Hg)	83.0	80.5	0.650	0.718
SBP nocturnal (mm Hg)	120.0	117.0	0.541	0.692
DBP nocturnal (mm Hg)	74.0	71.5	0.325	0.584
Weight (kg)	96.5	100.3	0.583	0.715
BMI (kg/m^2)	32.1	36.5	0.127	0.581
Hip circumference (cm)	117.5	117.5	0.776	0.827
Waist circumference (cm)	111.0	122.0	0.132	0.581
Waist to hip ratio	1.0	1.0	0.223	0.581
Body fat percentage (%)	43.2	43.6	0.477	0.636
HOMA-index	2.1	2.3	0.477	0.636
Insulin (mU/l)	9.7	8.2	0.290	0.581
Plasma glucose (mg/dl)	95.5	106.3	0.349	0.588
Hb-A1C (%)	5.7	5.9	0.152	0.581
Hb-A1C IFCC (mmol/mol)	38.6	40.8	0.152	0.581
Triglyceride (mg/dl)	135.6	153.1	0.280	0.581
Cholesterol (mg/dl)	230.0	193.3	1.000	1.000
HDL (mg/dl)	48.3	48.3	0.623	0.715
LDL(mg/dl)	148.8	121.8	0.451	0.636
LDL/HDL ratio	2.7	2.7	0.625	0.715
CRP (mg/l)	0.2	0.3	0.903	0.932
IL-6 (pg/ml)	2.5	2.1	0.200	0.581
Creatinine (mg/dl)	1.0	0.8	0.099	0.581
eGFR Cockroft-Gault (ml/min)	106.4	148.2	0.230	0.581
