# Supplementary material for: Fasting alters the gut microbiome reducing blood pressure and body weight in metabolic syndrome patients
Source: Nat Commun. 2021 Mar 30;12:1970. doi: 10.1038/s41467-021-22097-0 (PMC8010079; doi:10.1038/s41467-021-22097-0)
Supplement: Supplementary file 13 — Supplementary Data 10 [file 41467_2021_22097_MOESM13_ESM.pdf]

Supplementary Data 10. References for poorly characterized SCFA-producing microbes.

Microbe	Short Chain Fatty Acid produced	Reference
Eggerthella lenta	acetate	1
Hungatella hathewayi	acetate	2, 3
Eubacterium rectale	butyrate	4
Dorea longicatena	butyrate	5
Alistipes inops	acetate	6
Alistipes shahii	acetate, propionate	7
Pseudoflavonifactor	acetate	5
Acidaminococcus sp.	acetate, butyrate, (propionate?)	8,9

1. Wade WG, *et al.* The family Coriobacteriaceae: reclassification of Eubacterium exiguum (Poco et al. 1996) and Peptostreptococcus heliotrinreducens (Lanigan 1976) as Slackia exigua gen. nov., comb. nov. and Slackia heliotrinireducens gen. nov., comb. nov., and Eubacterium lentum (Prevot 1938) as Eggerthella lenta gen. nov., comb. nov. *International journal of systematic bacteriology* **49 Pt 2**, 595-600 (1999).

2. Kaur S, Yawar M, Kumar PA, Suresh K. Hungatella effluvii gen. nov., sp. nov., an obligately anaerobic bacterium isolated from an effluent treatment plant, and reclassification of Clostridium hathewayi as Hungatella hathewayi gen. nov., comb. nov. *International journal of systematic and evolutionary microbiology* **64**, 710-718 (2014).

3. Steer T, Collins MD, Gibson GR, Hippe H, Lawson PA. Clostridium hathewayi sp. nov., from human faeces. *Systematic and applied microbiology* **24**, 353-357 (2001).

4. Moens F, De Vuyst L. Inulin-type fructan degradation capacity of Clostridium cluster IV and XIVa butyrate-producing colon bacteria and their associated metabolic outcomes. *Beneficial microbes* **8**, 473-490 (2017).

5. Polansky O, Sekelova Z, Faldynova M, Sebkova A, Sisak F, Rychlik I. Important Metabolic Pathways and Biological Processes Expressed by Chicken Cecal Microbiota. *Applied and environmental microbiology* **82**, 1569-1576 (2015).

6. Shkoporov AN, *et al.* Alistipes inops sp. nov. and Coprobacter secundus sp. nov., isolated from human faeces. *International journal of systematic and evolutionary microbiology* **65**, 4580-4588 (2015).

7. Song Y, *et al.* Alistipes onderdonkii sp. nov. and Alistipes shahii sp. nov., of human origin. *International journal of systematic and evolutionary microbiology* **56**, 1985-1990 (2006).

8. Jumas-Bilak E, *et al.* Acidaminococcus intestini sp. nov., isolated from human clinical samples. *International journal of systematic and evolutionary microbiology* **57**, 2314-2319 (2007).

9. Rogosa M. Acidaminococcus gen. n., Acidaminococcus fermentans sp. n., anaerobic gram-negative diplococci using amino acids as the sole energy source for growth. *Journal of bacteriology* **98**, 756-766 (1969)
