# Supplementary material for: Fasting alters the gut microbiome reducing blood pressure and body weight in metabolic syndrome patients
Source: Nat Commun. 2021 Mar 30;12:1970. doi: 10.1038/s41467-021-22097-0 (PMC8010079; doi:10.1038/s41467-021-22097-0)
Supplement: Supplementary file 15 — Supplementary Data 12 [file 41467_2021_22097_MOESM15_ESM.pdf]

Supplementary Data 12. Classification of the probands of the Mesnage dataset according to their BP decrease after three months. A ≥ 5 mmHg decrease of the SBP at three months compared to baseline was considered to be significant.

Subject ID	SBP Baseline (mmHg)	SBP Follow-up, 3 months (mmHg)	ΔSBP Follow-up – Baseline (mmHg)	Classification (R=Responder, NR=Non-Responder)
1	115	117	2	NR
2	144	129	-15	R
3	133	122	-11	R
4	128	142	14	NR
5	112	120	8	NR
6	127	142	15	NR
7	134	130	-4	NR
8	137	127	-10	R
9	135	130	-5	R
10	138	135	-3	NR
11	122	104	-18	R
12	131	134	3	NR
13	131	123	-8	R
14	96	121	25	NR
15	158	138	-20	R
