# Supplementary material for: Fasting alters the gut microbiome reducing blood pressure and body weight in metabolic syndrome patients
Source: Nat Commun. 2021 Mar 30;12:1970. doi: 10.1038/s41467-021-22097-0 (PMC8010079; doi:10.1038/s41467-021-22097-0)
Supplement: Supplementary file 16 — Supplementary Data 13 [file 41467_2021_22097_MOESM16_ESM.pdf]

Supplementary Data 13

Reference	Intervention	Study Subjects and Design	Major Findings
Frost et al. **[3]**	Complex weight loss program	Low-calorie formula diet for six weeks, followed by nine weeks of food reintroduction in type 2 diabetic patients, N = 12.	• All participants lost weight during the program.<br>• Phylogenetic diversity increased parallel to weight loss.<br>• *Collinsella* abundance significantly decreased by end of the study compared to baseline.
Ozkul et al. **[4]**	Ramadan fasting	Ramadan fasting in healthy subjects, N = 9.	• Ramadan fasting did not alter alpha diversity (Shannon index) or phylogenetic diversity, but significantly changed the gut microbiome between baseline and after 29 days, according to unweighted UniFrac analysis.<br>• Ramadan fasting resulted in the enrichment of *Butyricicoccus*, *Bacteroides*, *Faecalibacterium*, *Roseburia*, *Allobaculum*, *Eubacterium*, *Dialister* and *Erysipelotrichici*, compared to baseline.
Louis et al. **[5]**	Complex weight loss program	Multidisciplinary weight-loss program lasting for 52 weeks followed by 12 months of follow-up in MetS/obese patients (N = 9, 7, respectively).	• At species level no significant difference could be observed between baseline and 24 months, yet at genera level *Akkermansia* was enriched significantly at 24 months compared to baseline.<br>• Patients with persistent weight loss success were enriched at baseline in *Alistipes*, *Pseudoflavonifractor*.
Velikonja et al. **[6]**	Beta-glucan supplementation for four weeks	Double blind, placebo-controlled study involving patients with MetS/ high risk for MetS, N = 43.	• Beta-glucan group significantly decreased the total cholesterol.<br>• Body weight significantly decreased in both groups.<br>• Fecal propionate increased in the beta-glucan group.<br>• In the beta-glucan group fecal diversity and richness (Chao, Shannon, respectively) decreased upon intervention.<br>• Cholesterol responders showed a higher baseline abundance of *Akkermansia muciniphila* and *Bifidobacterium* spp.
Roager et al. **[7]**	Whole grain vs. refined grain dietary intervention	Crossover clinical study with patients at risk of developing MetS with 8 weeks of intervention and 8 weeks of subsequent wash-out, N = 60.	• Intervention did not alter glucose homeostasis or fecal microbiome.<br>• Whole grain diet significantly decreased body weight compared to refined grain diet.<br>• The whole grain intervention induced weight loss, and decreased serum IL-6 and CRP.

Liu et al. **[8]**	db/db mouse experiment	28 days of intermittent fasting in rodents.	• Intermittent fasting decreased obesity-induced cognitive impairment and insulin resistance.<br>• Alpha diversity significantly increased and significantly changed beta-diversity<br>• Intermittent fasting improved Lactobacillus and the butyrate-producer *Odoribacter* abundances, meanwhile reduced the abundance of *Enterococcus*, *Streptococcus* and an unknown *Enterococcaceae*.
Kopf et al. **[9]**	Whole grain, fruit-vegetable supplementation vs. refined grain diet, 3 servings/day	Randomized, parallel arm study, involving overweight or obese patients, N = 49.	• Whole grain and fruit-vegetable diet both decreased the level of serum lipopolysaccharide binding protein, and increased alpha diversity.<br>• Fruit-vegetable and whole grain diet decreased serum IL-6 and TNFα levels, respectively, and these changes were correlated with the baseline microbiome composition.<br>• Fruit-vegetable group increased alpha diversity.<br>• BMI did not change significantly in any groups at the end of the study compared to baseline.
Guevara-Cruz et al. **[10]**	Complex weight loss, low saturated fat, 500 kcal/day dietary intervention	75-day long complex weight loss intervention program involving a MetS subpopulation of a cross-sectional study, N = 146.	• Intervention significantly decreased gut dysbiosis and increased the abundance of *Akkermansia muciniphila* and *F. parusnitzii* and with a reduction of *Prevotella/Bacteroides* ratio.<br>• The intervention improved the clinical phenotype; blood pressure, BMI, waist circumference, HOMA, HbA1c, LDL-c, glucose intolerance, LPS, BCAA decreased.
