# Supplementary material for: Fasting alters the gut microbiome reducing blood pressure and body weight in metabolic syndrome patients
Source: Nat Commun. 2021 Mar 30;12:1970. doi: 10.1038/s41467-021-22097-0 (PMC8010079; doi:10.1038/s41467-021-22097-0)
Supplement: Supplementary file 18 — Reporting summary [file 41467_2021_22097_MOESM18_ESM.pdf]

nature research

Corresponding author(s): Dominik N. Müller, Andreas Michalsen, Sofia K. Forslund

Last updated by author(s): Feb 3, 2021

Reporting Summary

Nature Research wishes to improve the reproducibility of the work that we publish. This form provides structure for consistency and transparency in reporting. For further information on Nature Research policies, see our Editorial Policies and the Editorial Policy Checklist.

Statistics

For all statistical analyses, confirm that the following items are present in the figure legend, table legend, main text, or Methods section.

n/a	Confirmed	
☐	☒	The exact sample size (*n*) for each experimental group/condition, given as a discrete number and unit of measurement
☐	☒	A statement on whether measurements were taken from distinct samples or whether the same sample was measured repeatedly
☐	☒	The statistical test(s) used AND whether they are one- or two-sided<br>*Only common tests should be described solely by name; describe more complex techniques in the Methods section.*
☐	☒	A description of all covariates tested
☐	☒	A description of any assumptions or corrections, such as tests of normality and adjustment for multiple comparisons
☐	☒	A full description of the statistical parameters including central tendency (e.g. means) or other basic estimates (e.g. regression coefficient) AND variation (e.g. standard deviation) or associated estimates of uncertainty (e.g. confidence intervals)
☐	☒	For null hypothesis testing, the test statistic (e.g. *F*, *t*, *r*) with confidence intervals, effect sizes, degrees of freedom and *P* value noted<br>*Give P values as exact values whenever suitable.*
☒	☐	For Bayesian analysis, information on the choice of priors and Markov chain Monte Carlo settings
☐	☒	For hierarchical and complex designs, identification of the appropriate level for tests and full reporting of outcomes
☐	☒	Estimates of effect sizes (e.g. Cohen's *d*, Pearson's *r*), indicating how they were calculated

Our web collection on statistics for biologists contains articles on many of the points above.

Software and code

Policy information about availability of computer code

Data collection	Diva (6.1.3)
Data analysis	Data analysis was performed by FlowJo 10.3, FCSExpress V6.02, GraphpadPrism 6, with the R packages FlowSOM (1.22.0), circlize (0.4.10), pheatmap(1.0.12), vegan (2.5-5), lmtest(0.9-37), orddom(3.1), DirichletMultinomial(1.32.0.), zoo(1.8-7), pipelines LotuS (1.62), NGLess framework (0.10), SILVA (v138), RTK (0.93.1), mOTUv2 (2.1), MOCAT2 (2.0.1), IGC gene catalog (0.5). Extensive data analysis is described in the corresponding sections of Methods.

For manuscripts utilizing custom algorithms or software that are central to the research but not yet described in published literature, software must be made available to editors and reviewers. We strongly encourage code deposition in a community repository (e.g. GitHub). See the Nature Research guidelines for submitting code & software for further information.

Data

Policy information about availability of data

All manuscripts must include a data availability statement. This statement should provide the following information, where applicable:

- Accession codes, unique identifiers, or web links for publicly available datasets
- A list of figures that have associated raw data
- A description of any restrictions on data availability

Data supporting the conclusions of this manuscript will be made available by the authors, without undue reservation, to any qualified researcher. The Python code for this analysis can be found online: https://github.com/fastingproject/Fasting_Paper_202086. Databases are to be found under the following links. KEGG: https://www.genome.jp/kegg/. SILVA: https://www.arb-silva.de. mOTU: https://motu-tool.org/, Mesnage dataset: https://www.ncbi.nlm.nih.gov/bioproject/PRJNA531091, IGC: https://db.cngb.org/microbiome/genecatalog/genecatalog_human/, Stool sequencing data: https://www.ncbi.nlm.nih.gov/bioproject/PRJNA698459

Field-specific reporting

Please select the one below that is the best fit for your research. If you are not sure, read the appropriate sections before making your selection.

- [x] Life sciences
- [ ] Behavioural & social sciences
- [ ] Ecological, evolutionary & environmental sciences

For a reference copy of the document with all sections, see nature.com/documents/nr-reporting-summary-flat.pdf

Life sciences study design

All studies must disclose on these points even when the disclosure is negative.

Sample size	For reference power calculation, we considered the case of testing for a significant difference between two time points in a paired test. With N = 27 fasting subjects, alpha threshold 0.05, and seeking 80% statistical power, we assessed the minimal effect size to achieve this power using the pwr R package with a paired t-test as the closest implemented equivalent to our nonparametric paired tests. The estimated standardized effect size (Cohen's d) is 0.56. This is within range of common moderate effects in microbiome studies, so our study is comparatively powered to similar studies for the sample size recruited.
Data exclusions	One patient was excluded from the fasting+DASH arm due to incomplete medical documentation.
Replication	Clinical parameters were assesed by standarized SOPs. Microbiome, immunome was evaluated by standardized and well-documented in-house pipelines. Samples were processed and analyzed as singlets.
Randomization	Participants were randomized into the intervention arms by block-randomization with randomly varying block length stratified by study center and intake/non-intake of antihypertensive medication by Random Allocation Software.
Blinding	Outcomes were evaluated by blinded scientists not involved in patient recruitment, allocation or treatment.

Reporting for specific materials, systems and methods

We require information from authors about some types of materials, experimental systems and methods used in many studies. Here, indicate whether each material, system or method listed is relevant to your study. If you are not sure if a list item applies to your research, read the appropriate section before selecting a response.

Materials & experimental systems

n/a	Involved in the study	
[ ]	[x]	Antibodies
[x]	[ ]	Eukaryotic cell lines
[x]	[ ]	Palaeontology and archaeology
[x]	[ ]	Animals and other organisms
[ ]	[x]	Human research participants
[ ]	[x]	Clinical data
[x]	[ ]	Dual use research of concern

Methods

n/a	Involved in the study	
[x]	[ ]	ChIP-seq
[ ]	[x]	Flow cytometry
[x]	[ ]	MRI-based neuroimaging

Antibodies

Antibodies used	The complete list of antibodies used along with their respective RRID, fluorochrome conjugation, manufacturer and dilution applied is listed in the corresponding Methods Table 1.
Validation	Validation of the antibodies in the on site laboratory was not performed and we refer to the respective specifcity information provided by the manufacturer. Miltenyi antibodies were validated by Miltenyi. eBioscience antibodies were validated by eBioscience. Biolegend antibody was validated by Biolegend.

Human research participants

Policy information about studies involving human research participants

Population characteristics	The population had a Caucasian-European background recruited in Germany. Characteristics of the Fasting+DASH and DASH arms were as follows. First values represent mean ± one standard deviation of the FASTING+DASH arm, second values represent the mean ± one standard deviation of the DASH arm.<br>Females/males: 23/12, 21/15<br>Age (year): 58±8, 62±8<br>Height (cm): 171±8, 171±9<br>Office SBP (mm Hg): 136±15, 138±16<br>Office DBP (mm Hg): 88±11, 88±9<br>24h ABPM SBP (mm Hg): 132±9, 131±9<br>24h ABPM DBP (mm Hg): 81±8, 81.4±7<br>24h ABPM MAP (mm Hg): 104±8, 104±7<br>24h ABPM peripheral resistance (mm Hg*s/ml): 1.4±0.1, 1.3±0.1<br>SBP day (mm Hg): 134±10, 133±10<br>DBP day (mm Hg): 83±9, 84±7<br>SBP nocturnal (mm Hg): 120±12, 121±10<br>DBP nocturnal (mm Hg): 71.5±8, 71.6±7<br>Weight (kg): 99±17, 96±17<br>BMI (kg/m2): 34±4.9, 33±4.7<br>Hip circumference (cm): 115±20, 113±17<br>Waist circumference (cm): 116±11, 114±12<br>Waist to hip ratio: 1.1±0.7, 1.0±0.2<br>Body fat percentage (%): 42±8, 39±10<br>HOMA-index: 2.8±2.1, 3.4±2.4<br>Insulin (mU/l): 10.4±6.4, 12.1±7.4<br>Plasma glucose (mg/dl): 105±20, 110±20<br>Hb-A1C (%): 5.8±0.4, 5.9±0.7<br>Hb-A1C IFCC (mmol/mol): 39.6±4.8, 41.2±7.4<br>Triglyceride (mg/dl): 166±106, 169±109<br>Cholesterol (mg/dl): 220±48, 222±54<br>HDL (mg/dl): 50±11, 51±10<br>LDL(mg/dl): 137±36, 140±45<br>LDL/HDL ratio: 2.8±0.7, 2.8±0.9<br>CRP (mg/l): 0.4±0.4, 0.3±0.3<br>IL-6 (pg/ml): 3.1±2.0, 2.8±2.2<br>Creatinine (mg/dl): 0.9±0.2, 0.9±0.2<br>eGFR Cockroft-Gault (ml/min): 120±39, 107±32
Recruitment	The population had a Caucasian-European background recruited in Germany. Patients were recruited from April 2014 until December 2015 by bulletins hung out at the Immanuel Hospital and at clinics of Charité. Information flyers were sent to cardiology and diabetology outpatient practices. Advertisments were published at the webpages of the Immanual Hospital and Charité Berlin and in local daily papers. In addition, the recruitment could already have introduced a selection bias toward patients being interested in fasting/dietary studies. As the study participants showed a high interest in the fasting intervention the allocated DASH participants were offered after successful completion of the study a cost-free subsequent fasting cycle.
Ethics oversight	The study was approved by the ethics committees of the Charité-Universitätsmedizin Berlin (approval number: EA4/141/13).

Note that full information on the approval of the study protocol must also be provided in the manuscript.

Clinical data

Policy information about clinical studies

All manuscripts should comply with the ICMJEguidelines for publication of clinical research and a completedCONSORT checklist must be included with all submissions.

Clinical trial registration	NCT02099968
Study protocol	The full protocol is in German language and it can be requested at andreas.michalsen@charite.de.
Data collection	Medical and clincal data was collected in 2014 and 2015 in Berlin, Germany. Secondary outcome measures were collected between 2014 and 2019 in Berlin and in Braunschweig, Germany.
Outcomes	Primary outcomes were defined systolic ABPM and HOMA index (time frame: 3 months). Secondary exploratory outcomes were assessed by multicolor flow cytometry and next generation sequencing investigating the microbiome and immunome composition. The complete list of secondary outcomes can be found at: https://clinicaltrials.gov/ct2/show/NCT02099968

Flow Cytometry

Plots

Confirm that:

- [x] The axis labels state the marker and fluorochrome used (e.g. CD4-FITC).
- [x] The axis scales are clearly visible. Include numbers along axes only for bottom left plot of group (a 'group' is an analysis of identical markers).
- [x] All plots are contour plots with outliers or pseudocolor plots.
- [x] A numerical value for number of cells or percentage (with statistics) is provided.

Methodology

Sample preparation	PBMCs were processed and analyzed as described previously18. Whole blood staining was performed using antibodies against major leukocyte lineages. Quantitative measurement was performed using a high throughput sampler (BD) and a BD FACS CantoII (BD). Cell numbers per µl blood were calculated as means from three independent measurements. Briefly, peripheral venous blood was obtained and mononuclear cells were isolated within 24 hours of collection by density gradient centrifugation using Biocoll and cryopreserved until further processing. Thawed cell aliquots were either labeled for extracellular antigens using fluorophore-conjugated monoclonal antibodies or CD4+ cells were selected (Miltenyi CD4+ Selection Kit). Cells (106) from CD4+ and CD4- fractions were placed onto U-bottom plates and re-stimulated for 4 hours at 37°C and 5% CO2 in a humidified incubator in a final volume of 200µl RPMI 1640 (Sigma) supplemented with 10% FBS (Merck), 100U/ml penicillin (Sigma), 100mg/ml streptomycin (Sigma), 50 ng/ml phorbol 12-myristate 13-acetate (PMA, Sigma), 250ng/ml ionomycin (Sigma) and 1.3µl/ml Golgistop (BD). After re-stimulation, cells were labeled with Life/Dead Fixable Aqua Dead Cell Stain Kit, for 405nm excitation (Invitrogen), followed by labelling with surface antigen-specific fluorophore-conjugated monoclonal antibodies. Cells were then fixated and permeabilized by FoxP3/Transcription Factor Staining Kit (eBioscience), and subsequently labeled with intracellular-antigen-specific fluorophore-conjugated monoclonal antibodies. Antibodies are listed in Materials and Methods Table 1. Samples were analyzed using the FACSCanto II multicolor flow cytometer (BD). Data analysis was performed using FlowJo 10.3 (FlowJo LLC) and FCSExpress V6.02 (De Novo Software) software. Absolute cell numbers were calculated using the relative percentage of cell population compared to a marker used in the whole blood staining.
Instrument	BD Canto II 4-2-2
Software	Diva 6 (BD), FlowJo 10.3 (FlowJo LLC) and FCSExpress V6.02 (De Novo Software)
Cell population abundance	Purity of the CD4+ enrichment was determined by flow cytometry detecting CD4+ cells after dead cell and doublet exclusion.
Gating strategy	Gating strategies started with the identification of cell populations on the linear SSC-FSC plot, followed by doublet and dead cell exclusion. Boundaries of specific positive and negative cell populations were determined previously using FMO, isotype and biological controls.

- [x] Tick this box to confirm that a figure exemplifying the gating strategy is provided in the Supplementary Information.
